# Recent Advances in Fluorescent Arylboronic Acids for Glucose Sensing

**DOI:** 10.3390/bios3040400

**Published:** 2013-12-10

**Authors:** Jon Stefan Hansen, Jørn Bolstad Christensen

**Affiliations:** Department of Chemistry, University of Copenhagen, Thorvaldsensvej 40, DK-1871 Frederiksberg C, Copenhagen, Denmark; E-Mail: jbc@chem.ku.dk

**Keywords:** continuous glucose monitoring, arylboronic acids, fluorescence, selectivity, immobilization, diabetes mellitus

## Abstract

Continuous glucose monitoring (CGM) is crucial in order to avoid complications caused by change in blood glucose for patients suffering from diabetes mellitus. The long-term consequences of high blood glucose levels include damage to the heart, eyes, kidneys, nerves and other organs, among others, caused by malign glycation of vital protein structures. Fluorescent monitors based on arylboronic acids are promising candidates for optical CGM, since arylboronic acids are capable of forming arylboronate esters with 1,2-*cis*-diols or 1,3-diols fast and reversibly, even in aqueous solution. These properties enable arylboronic acid dyes to provide immediate information of glucose concentrations. Thus, the replacement of the commonly applied semi-invasive and non-invasive techniques relying on glucose binding proteins, such as concanavalin A, or enzymes, such as glucose oxidase, glucose dehydrogenase and hexokinases/glucokinases, might be possible. The recent progress in the development of fluorescent arylboronic acid dyes will be emphasized in this review.

## 1. Introduction

Complications caused by change in blood glucose for patients suffering from diabetes mellitus can be avoided by stringent personal control offered by continuous glucose monitoring (CGM) and corresponding insulin administration. Damage to the heart, eyes, kidneys, nerves and other organs is, among other reasons, caused by high glucose concentrations, *i.e*., hyperglycemia. Proper glycemic control is, however, a difficult task, because a plethora of factors influence the glucose concentration, such as the timing of meals, the type and dosing of insulin, exercise, infections, *etc*. A key property of a continuous monitor is the constant surveillance and predictability of future blood glucose concentrations compared to intermittent monitoring, despite the fact that the latter displays a higher accuracy [1,2,3]. CGM can minimize unwanted fluctuations in blood glucose with a minimum operating effort in comparison to an intermittent monitor, which is invasive and inconvenient [4]. 

Semi-invasive glucose monitors provide significantly more comfort and less pain compared to their invasive counterparts [4,5]. These monitors are based on very sensitive fluorometric techniques, *i.e*., recording the change in emission intensity and fluorescence lifetime. The lectin concanavalin A (ConA) [6] enzymes, such as glucose oxidase (GOx) [7], glucose dehydrogenase [8], hexokinase/glucokinase [9], bacterial glucose binding proteins [10] and boronic acid derivatives [11] are usually employed.

The challenges to overcome for semi-invasive monitors are the demands for miniaturization, the long-term stability of the enzyme and transducer, oxygen deprivation, *in vivo* calibration, powering, short stability, baseline shift, safety and convenience [12,13,14,15,16]. In order to minimize discomfort associated with implementation, a very tiny size and proper sensor shape is required. Usually, inflammatory response to the implementable sensor in the subcutaneous tissue leads to the formation of fibrous capsules around the chemical sensors.

The elusive goal is the development of a reliable non-invasive glucose monitoring device. Non-invasive monitoring has been directed toward measurements in tears, saliva or sweat. However, the reliability of these approaches has been widely discussed [17,18,19]. 

## 2. Fluorescent Arylboronic Acid Sensors

Arylboronic acids are small and flexible molecules, which are capable of forming cyclic esters with 1,2-*cis*-diols and 1,3-diols, fast and reversibly in aqueous solution. Thus, fluorescent arylboronic acid sensors have gained increased interest over the last decades [11,20,21,22,23], due to the desirable properties, such as high sensitivity, detection through skin and less need for reference measurements. The main concepts in the design of fluorescent sensors are Photoinduced Electron Transfer (PET), Fluorescence Resonance Energy Transfer (FRET) and Internal Charge Transfer (ICT) [24,25,26,27,28].

Fluorescent systems can conveniently be divided into systems containing free fluorescent sensors and immobilized systems.

James and co-workers have been working on arylboronic acid-based sensors for more than two decades, exploiting the PET mechanism for sensing [29,30,31,32].

**Figure 1:**
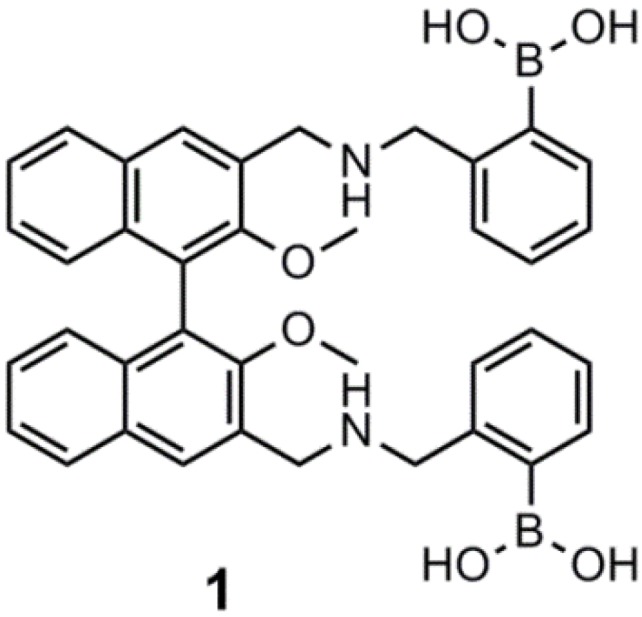
The chiral binol sensor, **1**, developed by James and co-workers [36].

The employed fluorescent dyes were based on anthracene [29,30], pyrene [31,32,33] and chiral binaphthols [34,35,36] coupled with diboronic acids to obtain superior d-glucose selectivity. Recent studies showed that the binol sensor was exhibiting a significant enantioselectivity for the binding of d-glucose at pH 6 (K_R_/K_S_ = 1.4:1) [36]. K_R_ is 2.01 × 10^2^ M^−1^, while K_S_ = 1.46 × 10^2^ M^−1^. Chiral discrimination was, however, not exhibited at pH 8, since d-glucose was bound with a similar strength by sensor **S1** and **R1**. K_R_ is 2.42 × 10^2^ M^−1^, while K_S_ = 2.32 × 10^2^ M^−1^ at pH 8. The λ_ex_ is 305 nm, and the λ_em_ is 374 nm. The binol sensor, **1**, is shown in Figure 1. The sensor, however, showed significantly higher binding affinity and chiral discrimination to important sugar alcohols, such as d-sorbitol, d-mannitol and xylitol. For d-sorbitol, K_R_/K_S_ = 1.09/5.88 at pH 6, and K_R_/K_S_ = 0.1 at pH 8. K_R_ is 1.09 × 10^3^ M^−1^, and K_S_ is 5.88 × 10^3^ M^−1^ at pH 6.0, while K_R_ is 1.13 × 10^3^ M^−1^ and 1.13 × 10^4^ M^−1^ at pH 8.0. All measurements were carried out in 52.1 w/w% methanol in water NaCl ionic buffer, due to the poor aqueous solubility of the binol sensor. 

James and co-workers also recently prepared two chiral diboronic acid sensors, **2** and **3**, both based on pyrene units [37]. Sensor **2** contains a naphthyl unit, while sensor **3** contains a *p*-methoxyphenyl unit. The most significant chiral discrimination is exhibited by sensor **2**, where K_R_ = 1,177 M^−1^, and K_S_ = 314 M^−1^ for binding of d-glucose. This gives K_R_/K_S_ = 3.7. K_R_ = 828 M^−1^ and K_S_ = 586 M^−1^ for the binding of d-glucose by sensor **3**, which gives K_R_/K_S_ = 1.4. A Job-plot of sensor **R2** indicates 1:1 binding stoichiometry.

**Figure 2:**
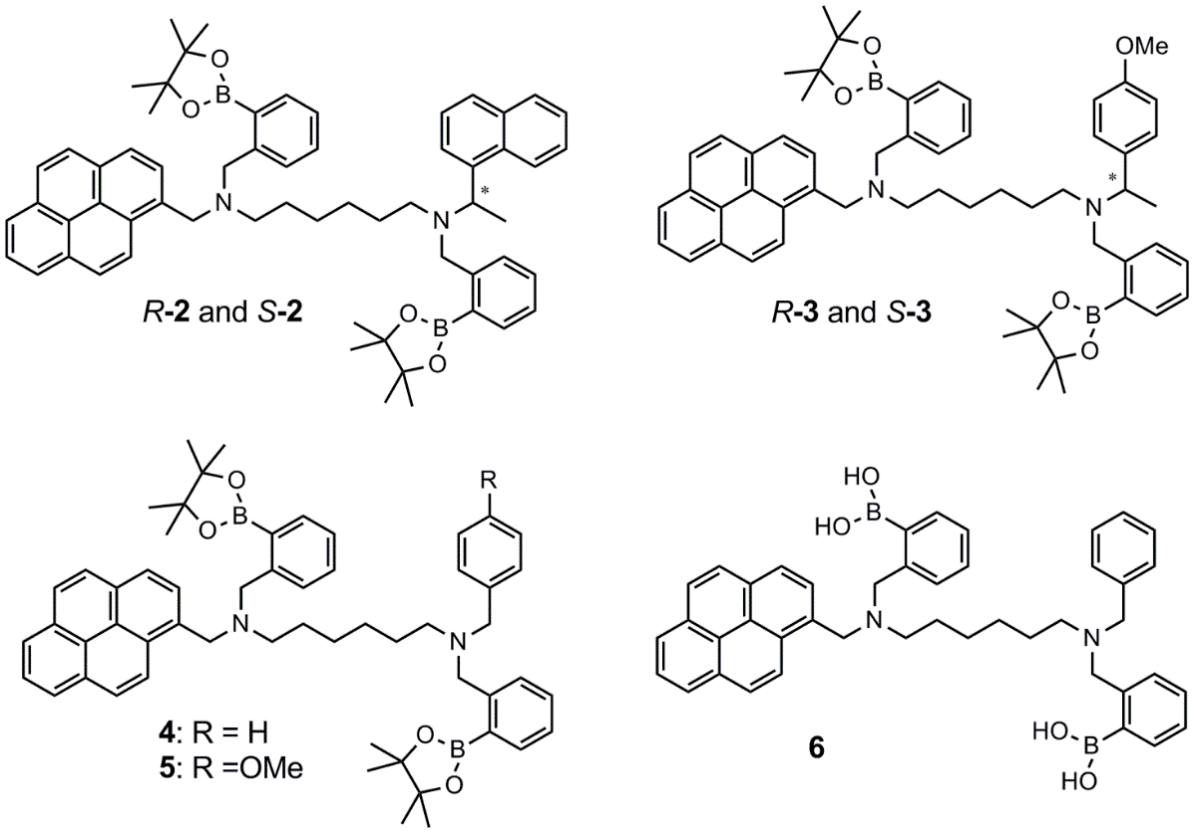
The aryl diboronic acid sensors, **2**–**6**, synthesized and tested by James and co-workers [37].

The d-fructose binding strength is very similar to the binding strength of d-glucose by sensor **2**. Furthermore, this sensor does not exhibit chiral discrimination. James and co-workers also developed the achiral sensors, **4**, **5** and **6**, which all exhibit a strong binding toward d-glucose. Binding constants are very similar, *i.e.*, K around 1,400 M^−1^ to 1,500 M^−1^. The d-fructose binding was found to be slightly weaker, with binding constants varying between 800 M^−1^ and 1,000 M^−1^. The sensors **2**–**6** are shown in Figure 2.

This study indicates the importance of built-in chirality in arylboronic acid sensors in order to obtain chiral discrimination in the binding of chiral molecules, such as saccharides. The sensors could be improved by adjusting the wavelength to the near-infrared area, enabling monitoring through skin. Improvement of the water solubility needs to be done in order to monitor physiological d-glucose in diabetes patients.

**Scheme 1:**
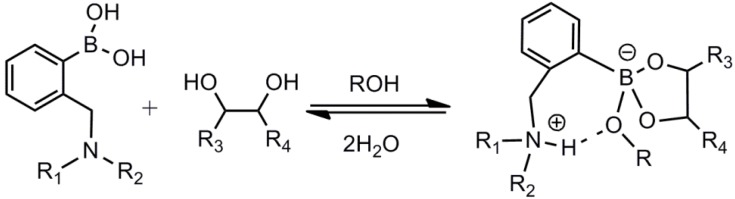
Formation of the corresponding boronate ester of an *o*-aminoalkyl phenylboronic acid by inclusion of a hydroxylic solvent molecule [38,39].

Research by Larkin *et al.* [38] and Collins *et al.* [39] has shown that the intramolecular dative B–N interaction responsible for the PET-mechanism almost is limited to systems without polar solvents. ^11^B-NMR and X-ray crystallography have revealed that a solvent molecule is inserted in all cases in a hydroxylic solvent, forming the zwitterionic complex shown in Scheme 1. Therefore, the PET-mechanism is predominantly facilitated by the extended protonation of the amino group, rather than B–N interaction, according to these studies. However, these observations are limited to sugars forming diester complexes with arylboronic acids, and not to, e.g., d-fructose binding, where triester formation is expected. 

**Scheme 2:**
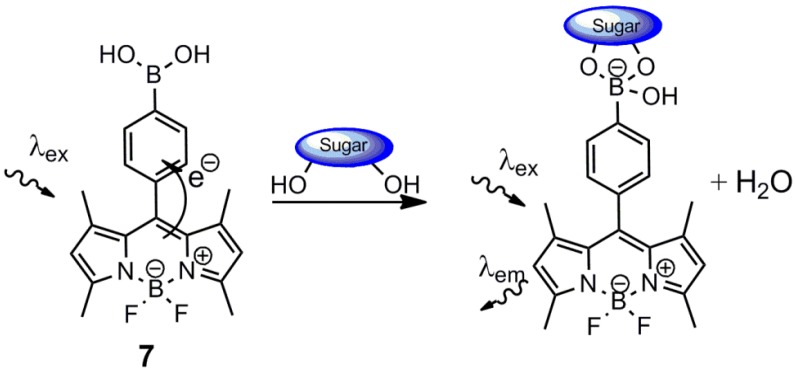
Proposed oxidative quenching of the BODIPY sensor, **7**, in the neutral form. The willingness to accept electrons decreases upon boronate formation, which increases the emission intensity [41].

Christensen and co-workers evaluated a fluorescent d-glucose sensor, **7**, based on the boron-dipyrromethene (BODIPY)-fluorophore, earlier reported by DiCesare and Lakowicz [40,41]. This sensor exhibited a significant increase in emission intensity at 510 nm upon d-glucose addition. However, this increase was found to depend on the buffered environment at physiological pH (7.4). The overall increase in intensity was highest in a 52.3 w/w% methanolic phosphate buffer, *i.e*., over 700%, whereas the increase was only around 30% in a phosphate buffer and a saline buffered solution. The increase in emission intensity was attributed to a reduced oxidative quenching upon formation of the aryl monoboronate. The calculated d-glucose binding constants were found to be buffer-dependent, with K = 19 M^−1^ (highest) and K = 10 M^−1^ (lowest) in the methanolic phosphate buffer and the saline buffer, respectively. The exhibited d-fructose binding strength was also buffer-dependent, displaying the weakest binding strength in the methanolic phosphate buffer with K =172 M^−1^ and the strongest complexation in the saline buffer with K = 455 M^−1^. This afforded a ratio K(d-glc)/K(d-frc) = 1:9 and K(d-glc)/K(d-frc) = 1:48. This row of experiments shows the buffer importance of binding affinity and underlines that binding constants are only comparable in similarly buffered environments. Thus, it is important to note that fluctuations in the blood environment may modulate d-glucose selectivity. The BODIPY sensor, **7**, is shown in Scheme 2, along with the proposed oxidative quenching.

**Scheme 3:**
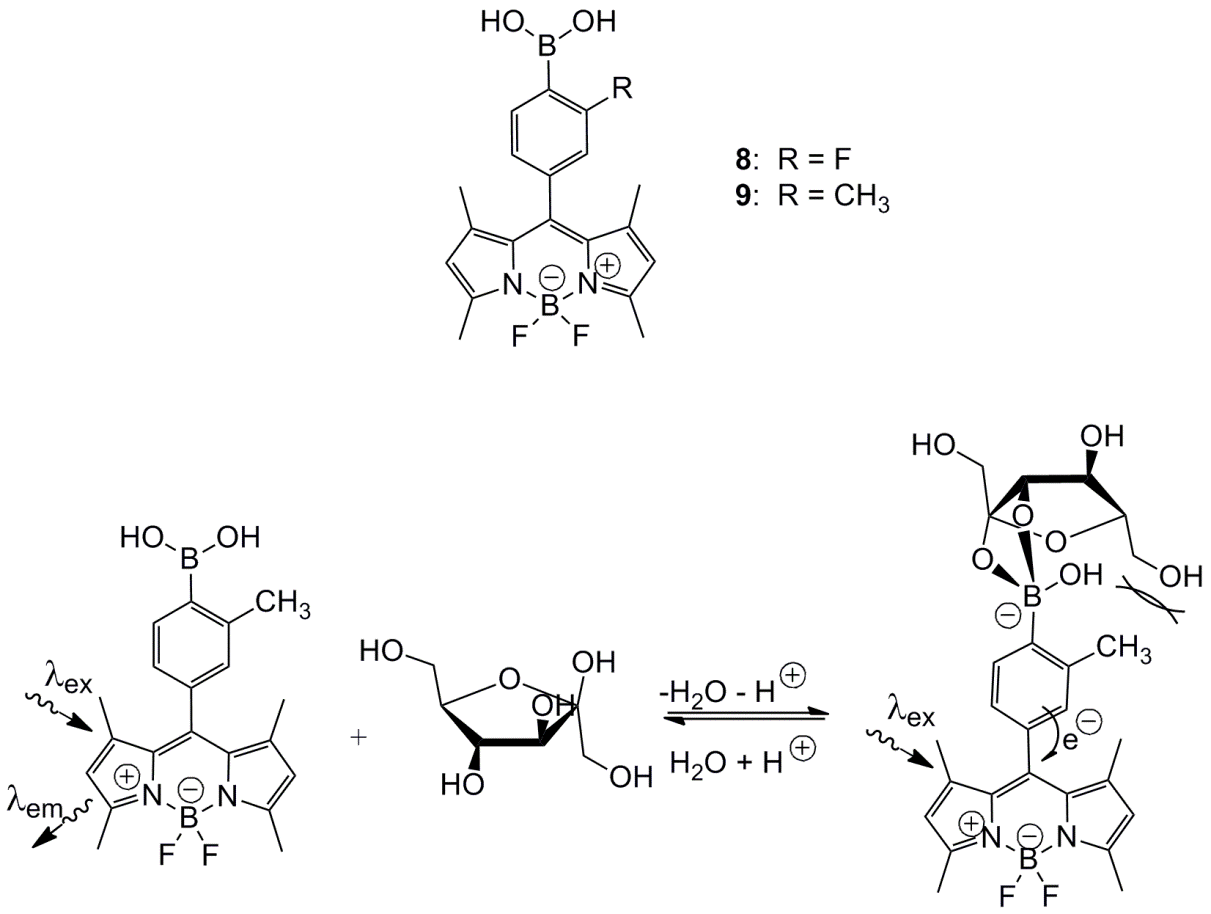
(**Top**) *ortho*-substituted aryl monoboronic acids, **8** and **9**, incorporated into a BODIPY fluorophore. (**Bottom**) Proposed reductive quenching for the diminished tridentate D-fructose binding of sensor **9**, caused by the presence of an *ortho*-positioned methyl substituent [42].

Christensen and co-workers also investigated the same type of BODIPY arylboronic acid, **8** and **9**, containing *ortho*-substituents [42]. This research showed that incorporation of fluorine in the *ortho*-position to the boronic acid created a sensor that was capable of binding d-glucose with a binding strength matching the mean value of the fluctuating blood glucose concentrations found in diabetics ([d-glc] varies between 2 mM and 30 mM). The association constant for d-glucose binding is very close to 60 M^−1^ in the three tested buffered solutions, *i.e.*, a saline buffer, a phosphate buffer and a 52.3 w/w% methanolic phosphate buffer. This assay robustness is very desirable for a future biological application of this sensor. The *ortho*-methylated BODIPY sensor, on the other hand, displayed a very weak d-glucose binding, *i.e*., K < 2 M^−1^, which underlines the importance of a sufficiently low pKa for proper d-glucose binding. Both sensors, **8** and **9**, are shown in Scheme 3. 

d-fructose selectivity was, in both cases, not reduced significantly by the presence of the *ortho*-substituent. However, the d-fructose binding affinity by the *ortho*-methylated sensor was reduced significantly by one order of magnitude [43]. The binding response was opposite of that observed for the *ortho*-fluorinated sensor, *i.e*., exhibition of reduced emission intensity. Earlier investigations have shown reduced d-fructose selectivity for small derivatives of phenylboronic acid containing *ortho*-substituents. The studies were performed by UV-Vis titrations with the colorimetric competitive assay Alizarin Red Sodium (ARS) and further rationalized by calculations at the B3LYP/6-31G(d)-level with the Gaussian [44]. The reported *ortho*-fluorinated d-glucose sensor could be modulated to bind d-fructose with a reduced affinity, and the wavelength of emission could be modulated towards the near-infrared area in order to monitor through tissue.

Singaram and co-workers have developed a lot of FRET-based viologen arylboronic acid sugar sensors. These sensors are capable of operating in aqueous solution at physiological pH and are highly sensitive to d-glucose at the physiological range [45,46,47,48,49,50,51]. Viologens are electron-deficient molecules, which enable them to act as quenchers for anionic dyes. Singaram and co-workers mixed their viologens with the water soluble anionic dye, pyranine. Pyranine can be excited by visible light, displaying a high fluorescence quantum yield, with λ_ex_ and λ_em_ of 461.8 nm and 511 nm, respectively. 

**Figure 3:**
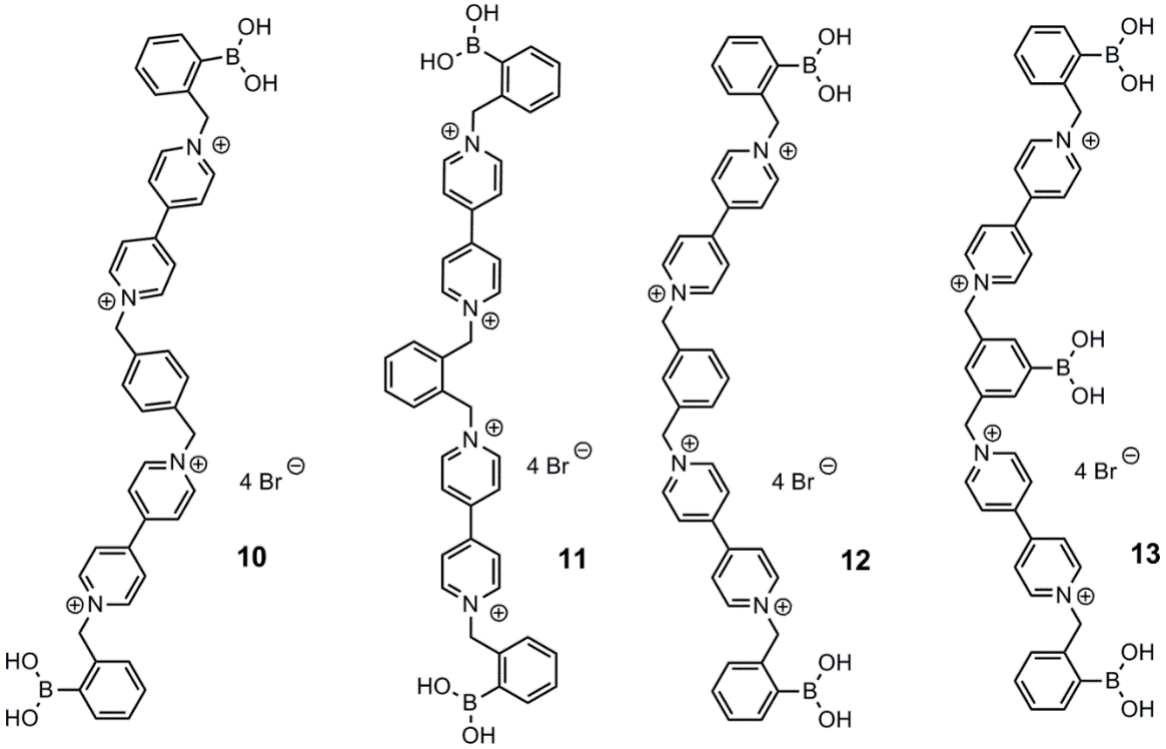
Viologens, **10**–**13**, developed and tested by Singaram and co-workers for the structural dependency on their quenching effect [48].

Recently, four quadrupole charged viologens, **10**–**13**, were developed in order to elucidate how the structural features influenced their respective quenching abilities [48]. The structures of **10**–**13** are depicted in Figure 3. The quenching ability was found to increase in the order **11** < **12** < **13** < **10**, which was ascribed to the decreasing tendency of pimer formation, caused by intramolecular stacking of the π-system in the viologens. In comparison to former published work [46], **10**–**13** are superior quenchers. This study indicates that the presence of the bisviologen core plays a greater part in the quenching rather than the charges. The highest d-glucose modulation was achieved with **13**. This was ascribed to a higher degree of negative charge in comparison to **10**–**12**. **10** and **12** were found to be far better quenchers than **11**. This phenomenon may be attributed to their poorer ability to form pimers. The highest quenching constant was obtained with **10**, followed by **13**, with K = 6.8 × 10^5^ M^−1^ and K = 4.4 × 10^5^ M^−1^, respectively. 

The viologens were further employed in glucose sensing hydrogels [49,50]. 

**Figure 4:**
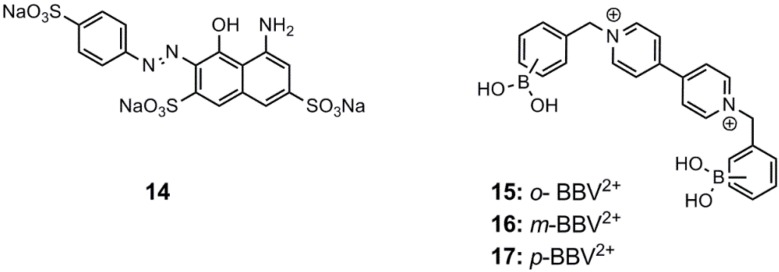
Singaram’s viologen arylboronic acids, **15**–**17**, employed by Feng and co-workers, using azo compound 14 as the fluorescent dye [52].

Feng and co-workers used the same two-component strategy with compounds **15**–**17** [52], earlier reported by Singaram and co-workers [45]. The employed fluorophore, azo compound, **14**, was found to emit fluorescence with λ_em_ = 585 nm, and maximum absorbance was recorded at λ = 530 nm. A significant d-glucose response from 0.4 mM to 150 mM was found. However, no binding constant was reported. Compounds **15**–**17** were also used together with CdTe quantum dots, where the increase in emission intensity at 635 nm for the quantum dots was monitored upon the addition of d-glucose to a mixture of quencher and CdTe quantum dots [53]. The quenching abilities were found to decrease in the order **15** > **16** > **17**. Thus, the highest increase by addition of d-glucose was found with **17**. The structures of the three viologens, **15**–**17**, and the azo compound, **14**, are shown in Figure 4.

Feng and co-workers also reported a dye for selective d-fructose recognition in the presence of other monosaccharides, such as d-mannose, d-galactose and d-arabinose, using pyranine as the fluorescent moiety [54,55]. 

Singaram’s and Feng’s systems are a great step in the development of glucose sensing devices. However, d-glucose selectivity is crucial and may be achieved by modulating the boronic acid distance, in order to bind in a cooperative manner. The wavelength should also be altered toward the near-infrared area in order to monitor through skin. The systems are, however, limited to *in vitro* monitoring, since dilution *in vivo* may be a problem for proper glucose readout. 

**Scheme 4:**
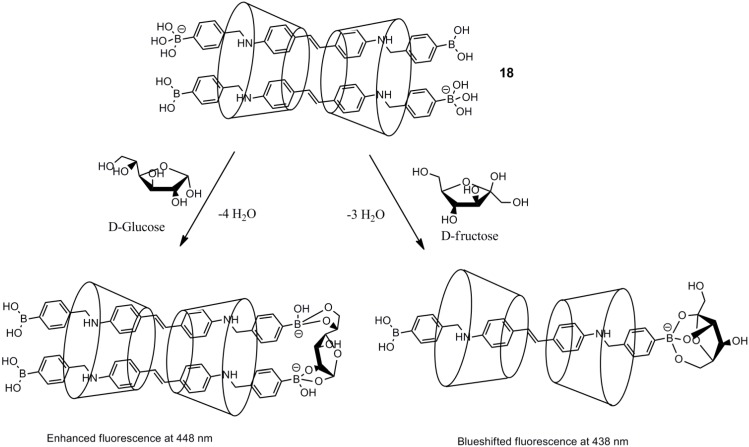
Interaction of d-glucose and d-fructose with the γ-cyclodextrin complex, **18**, forming two different inclusion complexes [56].

Wu *et al.* developed a stilbene-based aryl diboronic acid, which is capable of recognizing d-glucose selectively in comparison to d-fructose [56]. The sensoric system relies on the formation of a dimeric supramolecular complex of two stilbene derivatives within the cavity of a γ-cyclodextrin, **18**, which enables studies in aqueous media. The dimeric complex is stabilized due to bidentate binding of d-glucose by adjacent boronate units. The π-π-stacking upon dimer formation with the binding of d-glucose was observed to redshift the emission. However, the redshift was only modest, *i.e.*, λ_em_ = 438 nm → λ_em_ = 448 nm (λ_ex_ = 376 nm). d-fructose, which only stabilized the inclusion of a monomeric stilbene aryl diboronic acid within the γ-cyclodextrin cavity, did not give rise to a wavelength shift. The presence of the dimeric stilbene complex was proven by an induced exciton-coupled circular dichroism (CD)-signal. The d-glucose association constant was determined to be K = 1,048 M^−1^ at pH 10.5. The interaction of the γ-cyclodextrin complex with d-glucose and d-fructose is shown in Scheme 4.

d-glucose binding was found to be superior in a urine sample containing other saccharides, such as d-fructose, d-galactose and d-mannose, with a readout error less than 30%. This makes the sensor suitable for *in vitro* monitoring of d-glucose.

**Scheme 5:**
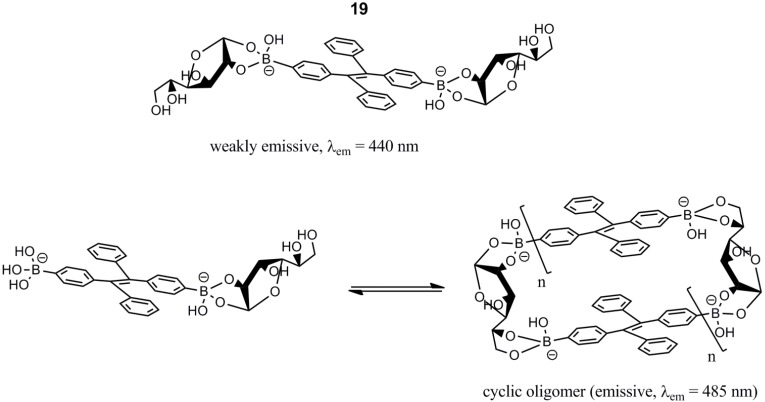
The weakly emissive 2:1 d-glucose-tetraphenylene diboronic acid complex of **19** (**top**). A significant redshift at 45 nm occurred, due to the formation of the emissive cyclic oligomer with d-glucose and the tetraphenylene diboronic acid sensor (**bottom right**). The linear oligomer is also redshifting in a similar manner [57].

Liu and co-workers reported a tetraphenylene-based fluorescent sensor, **19**, capable of forming oligomeric complexes with d-glucose [57]. Complex formation with d-glucose afforded exciplex formation, redshifting the emitted fluorescence significantly (λ_em_ = 440 nm → λ_em_ = 485 nm, λ_ex_ = 365 nm). The oligomeric complexes were formed as a consequence of the capability of d-glucose to form esters with two arylboronic acid units. This property is not present in other monosaccharides, such as d-fructose, d-galactose and d-mannose. Due to low aqueous solubility of the tetraphenylene sensor, the measurements were performed at high pH (10.5) in an aqueous carbonate buffer. The formed complexes are illustrated in Scheme 5.

Studies with samples all containing d-glucose and one of the saccharides, d-fructose, d-galactose and d-mannose, were performed. The affinity was the following order: d-fructose > d-galactose ≈ d-mannose > d-glucose. However, only d-glucose gave rise to an emission increase at 485 nm. This means that the reported sensor is d-glucose selective both in the presence and in the absence of the other mentioned saccharides. The association constant was, however, not reported for binding of d-glucose.

**Figure 5:**
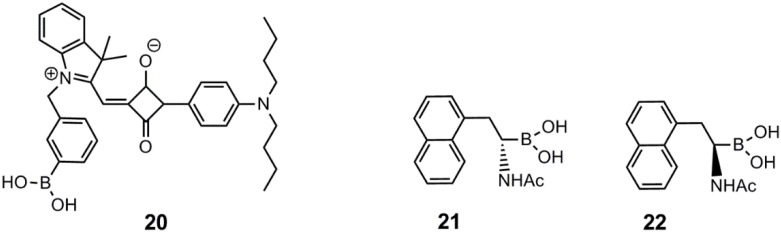
The near-infrared squarylium cyanine boronic acid dye, **20**, reported by Saito and co-workers [58], and the two enantiomeric α-amidoboronic acids, **21** and **22**, reported by Wang and co-workers [59].

Saito *et al.* reported a water soluble long-wavelength squarylium cyanine boronic acid dye, **20**, for the detection of monosaccharides [58]. This dye was found to exhibit superior d-fructose binding selectivity in comparison to other tested monosaccharides. The studies were conducted in an aqueous carbonate buffer at pH 10, with λ_ex_ = 630 nm and λ_em_ = 660 nm. An enhancement factor of 18 for the emission intensity was obtained in the presence of 20 mM d-fructose. The association constants for the binding of d-fructose and d-glucose was K = 628 M^−1^ and K = 8 M^−1^, respectively. This dye could be suitable for d-glucose monitoring through skin, due to the long wavelength of excitation and emission. The d-glucose selectivity must, however, be enhanced significantly and the d-glucose binding strength modulated towards physiological d-glucose levels. The reported dye, **20**, is shown in Figure 5. 

Wang and co-workers recently prepared water soluble, fluorescent and enantiomeric α-amidoboronic acids, **21** and **22**, attached to a naphthalene unit [59]. The two sensors did not show any chiral discrimination in the binding strength towards the tested monosaccharides. The apparent association constants at pH 7.4 of **21** with d-fructose, d-glucose and d-sorbitol were 55 M^−1^, 1.6 M^−1^ and 100 M^−1^, whereas the association constants of **22** with the same sugars were 46 M^−1^, 1.5 M^−1^ and 102 M^−1^, respectively. λ_ex_ = 280 nm, and λ_em_ = 334 nm. Sensor **21** and **22** are depicted in Figure 5. The change in fluorescence was attributed to the through-space interaction by the change in the hybridization of the boron core upon sugar binding. The performed studies also showed a significant decrease in quantum yield upon sugar binding for all saccharides in comparison to the obtained quantum yield without sugar. For sensor **21**, the yields were for free arylboronic acid, d-sorbitol-complex, d-glucose-complex, d-fructose-complex: 15.3%, 8.5%, 6.0% and 0.5% respectively. These values were about the same as recorded for **22**.

Several other articles describing the newest research in the field of fluorescent arylboronic acids for glucose detection have been published covering topics, such as supramolecular boronic acid complexes with cyclodextrins [60], benzoxazole alanine boronic acid derivatives [61], boronic acid anthraquinone derivatives via the glucose oxidase (GOx) enzymatic reaction [62], and the general timescale question of boronic acid binding with sugars in aqueous solution at physiological pH [63].

## 3. Immobilized Systems

**Figure 6:**
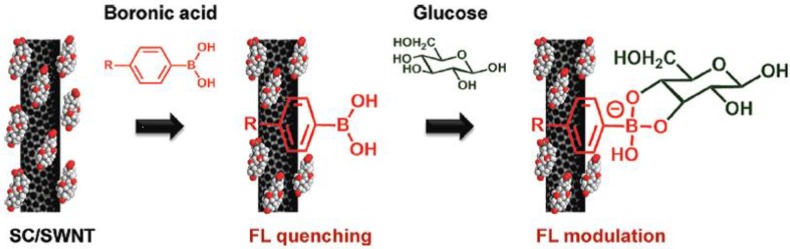
Binding of arylboronic acids to the surface of single-walled carbon nanotubes (SWCNTs) cause quenching of the near-infrared fluorescence, which reappears upon binding of glucose (Reprinted with permission from [64]. Copyright 2012 American Chemical Society).

**Figure 7:**
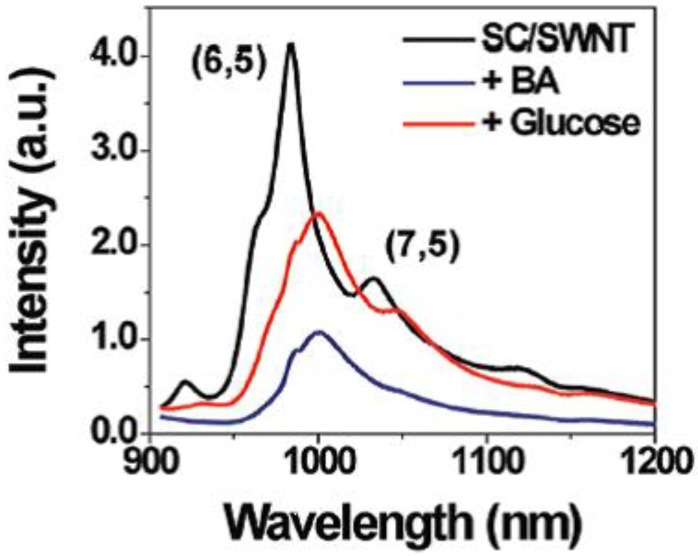
Modulation of the fluorescence upon binding of glucose to SWCNT-coated with 4-chlorophenylboronic acid (Reprinted with permission from [64]. Copyright 2012 American Chemical Society).

Immobilization of the sensing system offers many advantages for integration into a device, and this has been the topic for a number of research groups. Strano *et al.* [64,65] described a system consisting of aromatic boronic acids, which form stable complexes with single-walled carbon nanotubes (SWCNTs). The SWCNTs were solubilized in micelles in water by the surfactant, sodium cholate. SWCNTs are fluorescent in the near-infrared area, but upon binding of aromatic boronic acids, the fluorescence is quenched, and reappears upon the binding of carbohydrates, as shown in Figure 6. The altered emission spectra are depicted in Figure 7. The effect of fluorescence change was, however, dependent upon the structure of the boronic acid, and ensembles of 30 boronic acids in total were screened.

Qiu and co-workers [66] recently reported that combining an N,N′-bis(boronic acid) alkylated 4,4′-bipyridine with grapheme quantum dots gave a system that only became fluorescent after binding of glucose to the bis(boronic acid), which detached it from the quantum dot. They did not, however, investigate any selectivity for specific carbohydrates.

Manju and Sreenivasan [67] described a system-based gold nanoparticle coated with dextrane and functionalized with Rhodamine B and 3-aminobenzeneboronic acid, where binding of glucose to the boronic acid leads to emission from Rhodamine B. The system showed a good linear relationship between glucose concentration and emission, but the effect of interference from fructose was not investigated.

**Figure 8:**
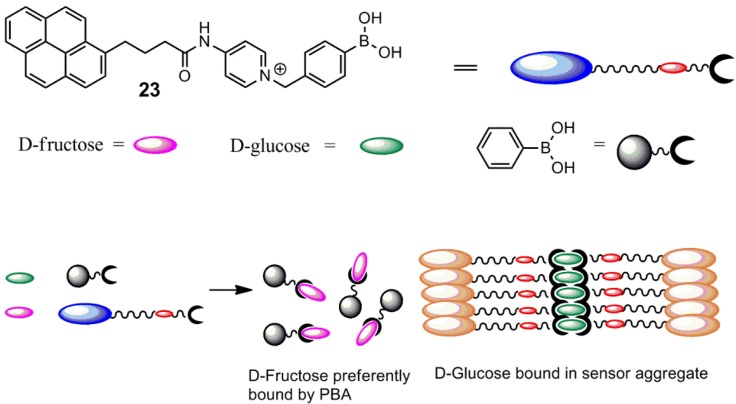
Cartoon representation of the different types of aggregates formed upon binding of glucose and fructose [68]. PBA, phenylboronic acid.

**Figure 9:**
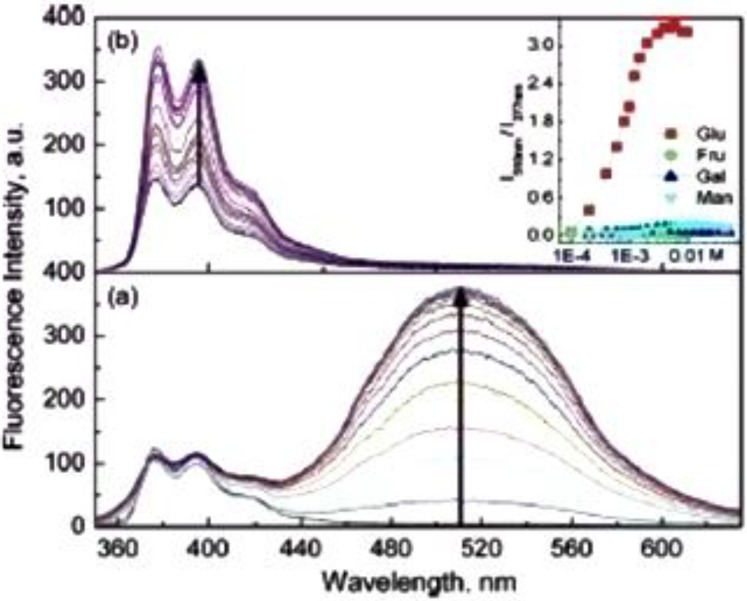
Fluorescence spectra recorded in a carbonate buffer (pH = 10) containing 2% methanol. (**a**) Glucose and (**b**) fructose, both in a concentration range from 0–10 mM. Insert I (a) shows the ratio between the excimer and monomer as a function of concentration and carbohydrate (Reprinted with permission from [68]. Copyright 2013 American Chemical Society).

An amphiphilic d-glucose selective aryl monoboronic acid sensor, **23**, containing a hydrophobic pyrene unit has been reported by James and co-workers [68]. They found that high d-glucose selectivity is facilitated by the formation of a 1:2 glucose-sensor complex, which is capable of forming aggregates in an aqueous buffer. d-glucose is capable of forming a boronate diester twice, which is exploited in this system. The positively charged sensor containing a pyridinium moiety becomes zwitterionic at boronate formation, which is facilitated by d-glucose binding. The sensor displays ratiometric properties, since the wavelength of fluorescence is redshifted significantly upon aggregate formation. The redshift is explained by the exciplex formation by the stacking of pyrene units, λ_em_ = 377 nm → λ_em_ = 510 nm. Phenylboronic acid (PBA) was used as a mask for d-fructose, since d-fructose is preferentially bound by PBA. This PBA addition enhanced the d-glucose selectivity further. Sensor **23** is shown in Figure 8, along with an illustration of the different binding mode of d-glucose and d-fructose. Thus, this system outlines the stoichiometry dependence, since there is a clear discrimination between glucose and fructose. The fluorescence spectra are shown in Figure 9.

The optimal conditions were found to be 0.10 mM of the sensor to make a compromise between sensitivity and the ease of aggregation control. The association constant for binding of d-glucose was found to be K = 1,378 M^−1^, whereas K = 353 M^−1^ was found for d-fructose binding. Despite the good d-glucose selectivity, the discovered sensor system is, however, not suitable for *in vivo* monitoring of blood glucose. The limitations are due to the dilution removing the aggregate. The wavelength of fluorescence is also not in the near-infrared area, which is a required property in order to minimize light absorption by the skin.

The potential of pyridinium containing arylboronic acids in polyol sensing was further explored through surface bound arylboronates and, e.g., modulation of exciplex formation in solution [69,70]. 

Incorporation of the complex between Alizarin Red S (ARS) and 2-formylbenzene boronic acid into aqueous micelles gives fluorescent micelles, where the fluorescence decreases upon adding glucose, due to the preferred binding of glucose to the boronic acid. This has been shown by Ngeontae and co-workers [71], who used simple surfactants, such as cetyltrimethylammonium bromide (CTAB), for the formation of the micelles. They observed an increased sensitivity upon using the micellar formulation, but the specificity of the system was fructose > xylose > glucose > maltose > lactose. Functional vesicles having boronic acids as part of the head groups have been investigated by Karpichev and co-workers [72], where vesicles were formed from an N-alkyl pyridium 3-boronic acid in water and binding of diols were measured using the Alizarin Red Sodium (ARS)-assay. The system, however, showed higher affinity for fructose than for glucose. 

**Figure 10:**
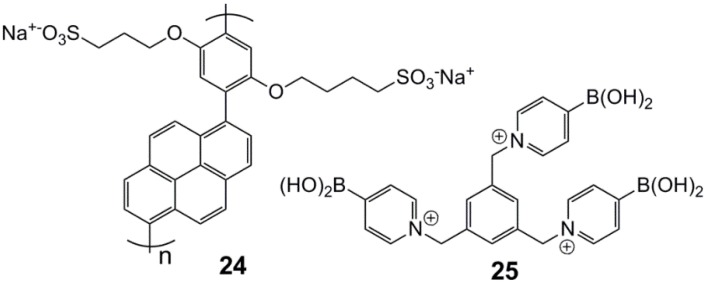
Poly(p-phenylene-pyrene)-sulfonate, **24**, and tris(pyridiniumboronic acid), **25** [73].

**Figure 11:**
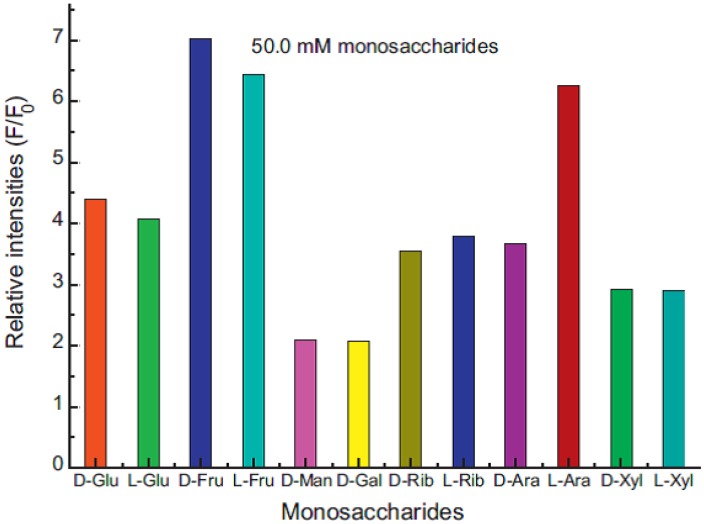
Fluorescence intensity at 467 nm for the system after the addition of 50.0 mM of monosaccharides at pH 7.4 buffer solution. Glu = glucose, Fru = fructose, Man = mannose, Rib = ribose, Ara = arabinose, Xyl = xylose (reproduced from [73]).

Polymer-based systems are interesting candidates for *in vivo* (diagnostics) and *in vitro* measurements of glucose, and a recent example of a system for diagnostics comes from Feng *et al.* [73], where a tridentate pyridinium boronic acid forms a non-fluorescent complex with a pyrene-containing polymeric polyelectrolyte (Figure 10 and Figure 11). The system shows some discrimination, but still, the main problem is achieving selectivity for glucose in the presence of fructose.

Another polymer-monomer-based system is the combination of an acrylamide copolymer containing benzene boronic acid units in combination with Bordeaux R, which is a red azo dye. This system, reported by Zhou and Li [74], formed a polymeric gel, where the addition of glucose leads to quenching of the fluorescence of the dye through the swelling of the gel.

Takeuchi and co-workers developed polyacrylamide-based boronic acid hydrogel fibers that were tested *in vitro*, demonstrating that it is possible to monitor the glucose concentration by transdermal measurements in a mouse model [75]. The fibers were implanted under the skin on the ears of the mice, showed only little inflammation and had a device lifetime of 140 days. 

## 4. Conclusion

In conclusion, fluorescent sensors based on arylboronic acids are very promising candidates for the elusive goal of performing noninvasive monitoring of d-glucose or other biologically important carbohydrates. This is due to the high sensitivity of fluorescence spectroscopy, which enables detection of very small changes in the analytical environment. 

The aryl monoboronic acid sensors generally favor binding of d-fructose over d-glucose, which is attributed to the different binding mode, *i.e.*, tridentate d-fructose binding *versus* bidentate d-glucose binding. BODIPY sensor **8** showed binding of d-glucose at the physiological level, but d-fructose was bound significantly more strongly [42]. Superior d-glucose selectivity is achieved by complexation with aryl diboronic acid sensors. Aryl diboronic acid dyes are usually saturated at physiological d-glucose levels, *i.e.*, [d-glc] ≈ 5 mM, and these sensors are usually not water soluble, due to the large hydrophobic scaffold, which is required in order to place the boronic acid units in an appropriate cooperative distance. Aryl diboronic acid equivalents have been formed by aggregate formation with d-glucose [68] or by inclusion of lipophilic aryl monoboronic acids into cyclodextrin cavities [56]. Fluorescent properties, such as wavelengths of excitation and emission, and potential fluorescence lifetime experiments are to be optimized in future systems. The emphasis may also be on systems with fewer tendencies to photobleach or oxidize. Proper glucose-readout may be monitored through skin. Thus, a suitable glucose sensor may be tattooed into the subcutaneous layer of human skin in order to perform optimal regulation of blood glucose in diabetics, providing safety and comfort. The dye must be near-infrared to avoid skin absorption of light; otherwise, an optical fiber is needed, which is painful, due to the surgical procedure of implantation. 

Ultimately, blood glucose can be determined noninvasively in the case of a proper tear glucose/blood glucose correlation. 
